# A Digital Capabilities Dataset From Small- and Medium-Sized Enterprises in the Basque Country (Spain)

**DOI:** 10.3389/fpsyg.2020.587949

**Published:** 2021-01-28

**Authors:** Nekane Aramburu, Klaus North, Agustín Zubillaga, María Paz Salmador

**Affiliations:** ^1^Deusto Business School, University of Deusto, San Sebastián-Bilbao, Spain; ^2^Wiesbaden Business School, Wiesbaden, Germany; ^3^Orkestra-Basque Institute of Competitiveness & University of Deusto, San Sebastián-Bilbao, Spain; ^4^Department of Business Organization, Universidad Autónoma de Madrid, Madrid, Spain

**Keywords:** dynamic capabilities, digital capabilities, digital maturity, SMEs, Basque Country, Spain

## Introduction

Sustainable competitiveness and growth of small- and medium-sized enterprises (SMEs) are increasingly determined by their capability to make use of digital technologies [EU (European Commission), 2018b] and tie into a digital ecosystem (Pelletier and Cloutier, 2019). Surveys (Tarutea and Gatautis, 2014; Bouwman et al., 2019; Shettima and Sharma, 2020) prove that the digitalization has a positive effect on the performance of SMEs. This includes dimensions such as growth, market value, and profitability as well as social and environmental performance and satisfaction; 46% of firms that participated in a survey of the European Digital Transformation Scoreboard report a medium-to-large increase in their annual turnover over the last 3 years following the adoption of technology [EU (European Commission), 2018a]. Many SMEs, however, are lagging behind in digital transition (OECD, 2017). According to a report by the Digital Innovation Hubs Working Group (2018), only 17% of SMEs have successfully integrated digital technologies into their businesses, compared with 54% of large companies. They lack resources and capabilities or suffer from inertia, which hampers opportunities (Cenamora et al., 2019). In the emerging highly interconnected and collaborative forms of value creation, the capacity to connect better to an integrated business network will be important to stay competitive (Rehm and Goel, 2017; EU (European Commission), 2014).

SMEs comprise three different categories of enterprises, namely, micro-enterprises, small enterprises, and medium-sized enterprises (see Table 1). To classify firms, the official European definition of SMEs considers three different factors: level of employment, level of turnover, and size of balance sheet.

**Table 1:** Definition of SMEs.

**Company Category**	**Employees**	**Turnover**	**Balance sheet total**
Micro	<10	<€2 million	<€2 million
Small	<50	<€10 million	<€10 million
Medium sized	<250	<€50 million	<€43 million

*Source: European Commission Recommendation dated 6 May 2003 regarding the definition of micro-enterprises, small-sized enterprises, and medium-sized enterprises (2003/361/EC), Official Journal of the European Union, L 124/36, 20 May 2003*.

According to the EU (European Commission) (2018b) overall, in 2017, SMEs in the EU accounted for 99.8% of all EU-28 nonfinancial business sector enterprises, two–thirds of total EU-28 employment (66.4%), and slightly less than three–fifths (56.8%) of the value added generated by the nonfinancial business sector. Micro-SMEs are by far the most common type of SME, accounting for 93.1% of all enterprises.

SMEs are a highly diverse group of enterprises that also condition how they approach digitalization (OECD/UN ECLAC, 2012; Neirotti et al., 2018). For example, approaches differ in case of Industry 4.0 adoption (Matt and Rauch, 2020) or the integration in platform ecologies (Gierlich-Joas et al., 2019). A common denominator, however, is the need to integrate, build, and reconfigure internal and external resources in order to adapt to rapidly changing environments (North and Varvakis, 2016). These dynamic capabilities take the form of skills, processes, procedures, organizational structures, and decisions that motivate and promote the detection (sensing) and capture (seizing) of opportunities in order to reconfigure (transform) their capabilities (Teece, 2007). As several studies show, the development of dynamic capabilities impacts SME performance and growth (He and Wong, 2004; Lubatkin et al., 2006; Macpherson and Holt, 2007; Protogerou et al., 2008; Sunday and Vera, 2018) and is vital for implementing Industry 4.0 approaches (Garbellano and Da Veiga, 2019) and digitalization (Matarazzo et al., 2021).

However, currently, there is a limited understanding of how SMEs are approaching digitalization from a dynamic capabilities perspective. Garzoni et al. (2020) introduce a four-level approach of engagement of SMEs in the adoption of digital technologies, namely, digital awareness, digital enquirement, digital collaboration, and digital transformation, hence the need to map adoption and learning paths of these firms. For this mapping, digital maturity models or frameworks can provide guidance (Valdez de Leon, 2016, Williams et al., 2019). The DIGROW digital maturity framework (North et al., 2020) is grounded on the microfoundations of dynamic capabilities (Teece, 2007) and therefore allows to link digitalization to organizational capabilities. Based on this framework, a questionnaire has been built and applied to a sample of 380 SMEs from the Basque region (Spain). In the following section, we describe the framework, the structure of the questionnaire, the data collection process, and the content of the database built as a result of this process.

## Method

### The DIGROW Framework of Digital Maturity

The DIGROW framework of digital maturity (North et al., 2020) aims at companies to assess their digital maturity level, and the capabilities associated with each level of maturity, which could support their digitally enabled growth. The framework is grounded in dynamic capabilities theory. In the explanation of microfoundations of dynamic capabilities, Teece (2007) described his constituent capacities: “For analytical purposes, dynamic capabilities can be disaggregated into the capacity (1) to sense and shape opportunities and threats, (2) to seize opportunities, and (3) to maintain competitiveness through enhancing, combining, protecting and, when necessary, reconfiguring the business enterprise's intangible and tangible assets” (Teece, 2007: 1319). Pavlou and El Sawy (2011), based on their empirical research, proposed four steps of dynamic capabilities development—sensing, learning, integration, and coordination—thus highlighting the importance of managing knowledge and learning to cope with turbulent and disruptive environments (North and Varvakis, 2016).

A particular shortcoming in SMEs is that owners and managers are aware of growth potentials; however, they tend to lack an explicit strategy, and if they have one, they do not communicate that strategy to employees (North et al., 2016). Therefore, in the DIGROW framework, an intermediate step is inserted between Teece's “sensing” and “seizing,” the step of strategy development and communication, which is related to Pavlou and El Sawy's (2011) learning and integration. Thus, the “DIGROW” framework considers four capacities:

1. *Sensing digitally enabled growth potentials:* searching for digitally enabled growth opportunities, understanding and developing digital customer needs, sensing technology-driven opportunities, and use of external sources for digital innovation.
2. *Developing a digitally enabled growth strategy and mindset:* Digitally enabled growth strategy, digital leadership, digital mindset (attitudes and behaviors), and empowered employees.
3. *Seizing digitally enabled growth potentials:* Digitally enabled business models, digital market presence, digital customer experience, and agile implementation/deployment of digitalization initiatives.
4. *Managing resources for digital transformation:* Digital skills and learning, digital processes, digital technology and security, and digital investments.

Each of these capacities is assessed at six levels described by an anchor statement. A pretest in selected firms (North et al., 2019) revealed that these six levels would allow a sufficient degree of differentiation. As mentioned above, a self-assessment questionnaire has been developed based on this framework. This is shown in the Appendix.

### Data Collection

The companies subject to study are small- and medium-sized enterprises (SMEs) from the Basque region in Northern Spain, which, according to the definition of SME proposed by the European Union, comprise between 10 and 249 employees.

The questionnaire has been addressed to 7,040 firms in cooperation with regional business associations between July and November 2018 and was answered by the chief executive officer (CEO) or the information technology (IT) manager in each firm. The number of SMEs that responded to the survey amounted to 540 (response rate 7.67%). After only partially completed or invalid questionnaires were eliminated, the final sample consisted of 427 companies. As for company size, 47 firms were micro-enterprises (i.e., <10 employees), 220 were small firms (i.e., between 10 and 49 employees), and 160 were medium-sized firms (i.e., between 50 and 249 employees).

Regarding composition of the sample according to industries, 133 firms belong to the manufacturing sector and 24 to commerce, 198 companies are distributed among different types of services (i.e., education, health services, insurance, information services, transport, and professional services), and 25 companies belong to the building sector.

### Database Content

Based on the data collected, we built a database in which the structure and content are described in this section.

The database is structured in rows and columns. Each row contains the information related to each firm (in total, 427 firms). On the other hand, the columns include the following information:

– Industry where the company operates. Industries are codified according to NACE (A21) classification.
– Range of employees per company. We consider these data to categorize the firm as a micro-enterprise (range = 0–9 employees), a small company (range = 10–49 employees), or a medium-sized company (range = 50–249 employees).
– The level of maturity reported by each firm regarding each question referred to firm's digital capacities (16 questions/capacities in total). We distinguish six maturity levels. Levels 0 and 1 correspond to a low degree of a firm's digital maturity; levels 2 and 3 correspond to a medium degree of a firms' digital maturity; and levels 4 and 5 refer to a high degree of a firm's digital maturity (see the Appendix). These level values are reported in the database and ranged from 0 to 5.

## Data Usage

The data contained in this database can be analyzed for different purposes.

The main purpose is to assess the level of digital maturity of each company. In order to obtain an overall picture of the level of digital maturity, we have carried out a descriptive analysis, in particular, a frequencies analysis. We used the software IBM SPSS (version 26.0).

First, a frequency analysis for the whole sample allows us to obtain the number and percentage of companies that rated each one of the levels of digital maturity for each one of the capacities considered. In other words, for each capacity, we could know how many firms attain a particular level of maturity. Based on this, we could conclude if the maturity level achieved by each company regarding each capacity was low, medium, or high. The results of the frequency analysis are as follows: Regarding sensing potential opportunities for digital growth, a high number of companies are able to search and identify growth opportunities (77%), and 28.2% of firms work actively on their identification. Nevertheless, only 24% identify growth opportunities systematically.

As far as developing a digitally based growth strategy and mindset is concerned, while many companies understand the relevance of digitalization, they are not able to develop strategies aimed at taking advantage of the growth opportunities opened by digital technologies. Only 18% of companies define a digitally enabled growth strategy, and 15% update their strategy, taking into account different facets of digitally enabled growth.

In terms of seizing digitally enabled growth potentials, 31.6% of companies do not have a digitally based business model, while 22% of firms claim to have started to change some components of their business models. Finally, only 14.4% of companies systematically adapt their business models or create new business models to promote a digitally enabled growth.

With regard to managing resources for digital transformation, approximately a quarter of firms (26%) consider that investment to develop digital skills is low. And only 6.7% of companies claim to possess the necessary digital skills. On the other hand, almost a quarter of firms (24%) claim to achieve a medium level of investment in digital transformation initiatives, while only 9.5% of companies consider they invest a lot in digitalization.

Second, we also run a correlation analysis. Observing the correlation matrix, we find that the highest correlated variables are the following: digitally enabled growth strategy highly correlates with digital leadership (0.76) and a digitally based business model (0.70). Moreover, a digitally based business model correlates with digital market presence (0.70) and digital customer experience (0.72). Digital customer experience also highly correlates with digital skills and learning (0.74). Finally, there is a high correlation between digital skills and learning and agile implementation of digital initiatives (0.72). On the other hand, we run a factor analysis, but this does not show relevant results, since it only discriminates one factor, probably due to the high extant correlation among most of variables (i.e., capacities).

Third, we carried out a regression model analysis to explore the relationship between a digitally enabled growth strategy as the dependent variable and digital mindset, digital leadership, and empowered employees as the independent variables (Aramburu et al., 2020). The results of the regression model test show that the relationship between each independent variable and the dependent one is significant in all cases at a significance level of 95% (*p* < 0.05; see Table 2). Therefore, digital leadership, digital mindset, and the fact of having empowered employees who deploy digital initiatives have a positive and significant influence on digitally enabled growth strategy. In addition, digital leadership is the most relevant capacity influencing digitally enabled growth strategy (β = 0.533), followed by digital mindset (β = 0.287) and empowered employees (β = 0.151).

**Table 2:** Regression model (coefficients and significance).

**Model**	**Non-standardized coefficients**		**Standardized coefficients**	***t***	**Sig**.
	**B**	**Standard error**	**Beta**		
(Constant)	0.418	0.183		2.288	0.023
Your company has a digital leadership	0.533	0.051	0.522	10.522	0.000
Your company has developed a digital mindset	0.287	0.066	0.201	4.343	0.000
Your company empowers employees to experiment with digital initiatives	0.151	0.049	0.144	3.107	0.002

*Note: Dependent variable: Your company has a digitally enabled growth strategy*.

Finally, further analyses have been carried out with the aim of testing the role of firm's size. With this purpose, a mean comparative analysis has been carried out comparing small- and medium-sized companies (i.e., between 50 and 249 employees) and big firms (i.e., between 250 and 500 employees), showing that there is a significant difference according to the size of the firm only in the case of eight capacities included in the framework over a total of 16 (i.e., use of external sources for digital innovation, digital leadership, empowered employees, digitally enabled business models, digital market presence, digital customer experience, agile implementation/deployment of digitalization initiatives, and digital skills and learning). Therefore, we conclude that the firm's size affects digital maturity, but this effect is not extremely relevant.

## Limitations and Future Research Avenues

Additional types of data analysis could be carried out. For instance, and considering the data contained in the database, further regression analyses might be carried out in order to explore the relationships among different sets or combinations of capacities. One potential area of interest to explore would be to analyze which factors can influence the digitalization processes, such as digital skills, digital investments, and digitally enabled growth strategy. Another future avenue could be to explore how digitally enabled business models are influenced by the digitally enabled growth strategy, digital investments, and the digital mindset. Finally, a future relevant path for research is opened regarding the impact of coronavirus disease 2019 (Covid-19) in digital transformation of SMEs, in terms of both firms' capabilities and also firms' characteristics (e.g., size and industry). The pandemia is catalyzing digitalization processes in many companies; thus, it would be interesting to explore what is happening in SMEs.

To conclude, the database has some limitations, such as it only includes data of SMEs from a particular geographical setting (i.e., Basque region in Spain). Moreover, it only refers to SMEs, not including data of big companies. Regarding the industries represented, the dataset is quite complete since it contains data of SMEs belonging to all industries in the region. Finally, another limitation is that the database does not contain data regarding the companies' performance (i.e., revenues or growth rate). This might be completed in the future collecting additional data about performance.

## Data Availability Statement

The datasets presented in this study can be found in online repositories. The names of the repository/repositories and accession number(s) can be found in the article/supplementary material. The dataset is available here: http://data.orkestra.deusto.es/es/dataset/digrow-basque-sme.

## Author Contributions

NA has led the writing process of this manuscript. KN has led the development of the digital maturity framework referred to in this paper (i.e., DIGROW framework). AZ has led the data collection process and database building. MPS has contributed to the writing of a part of the article. All authors contributed to the article and approved the submitted version.

## Conflict of Interest

The authors declare that the research was conducted in the absence of any commercial or financial relationships that could be construed as a potential conflict of interest. The handling editor declared a past co-authorship with one of the authors MPS.
