# Supplementary material for: A Digital Capabilities Dataset From Small- and Medium-Sized Enterprises in the Basque Country (Spain)
Source: Front Psychol. 2021 Jan 28;11:587949. doi: 10.3389/fpsyg.2020.587949 (PMC7875880; doi:10.3389/fpsyg.2020.587949)
Supplement: Supplementary file 1 [file Presentation_1.pdf]

# Appendix: Questionnaire based on DIGROW framework

## I. Sensing digitally enabled growth potentials

| Dimension | Level 0 | Level 1 | Level 2 | Level 3 | Level 4 | Level 5 |
|---|---|---|---|---|---|---|
| **Searching for digitally enabled growth opportunities** | Digitalization is rather a threat than an opportunity for us | We are planning to identify opportunities | We have started<br><br>to look for digital enabled growth opportunities | We have spotted some opportunities | We identify digital enabled growth opportunities, but not systematically | Our company identifies systematically digitally enabled growth opportunities |
| **Understanding and developing digital customer needs** | No understanding of digital customer needs | We are planning to talk to customers and analyze digital needs | We have talked to some customers about digitalization | We have analyzed some customer segments | We have a clear understanding how our most important customer segments are changing in the digital environment and what their needs are | There is genuine and systematic understanding of how each of our customer segments are changing in the digital environment, what their needs are, and how to address them |
| **Sensing technology driven opportunities** | No digital / technological innovation | We react on digital technology of competitors | We observe new digital technologies/ applications<br><br>And how they could serve us | We appropriated some new digital technology | We analyze how some technology driven opportunities can create value for specific customer segments and we address them properly | Our company takes a systematic and proactive approach to technology driven product/service innovation in the digital environment |
| **Use of external sources for digital innovation: (potential) customers, universities, research centers, "the crowd", partners in the "eco-system"** | No use of external sources | We are planning to collect ideas from customers and other stakeholders (providers, universities, etc.) | We have started to use external sources from different players | External sources have contributed to identify innovation opportunities | We collaborate regularly with some external partners to search for and develop digitally enabled growth opportunities | We practice a systematic and proactive open innovation approach: Invite customers/ potential clients to provide feed-back and ideas via digital platforms (Crowdsourcing), we collaborate intensively with further external sources such as providers and universities |

## II. Developing a digitally enabled growth strategy and mindset

| Dimension | Level 0 | Level 1 | Level 2 | Level 3 | Level 4 | Level 5 |
|---|---|---|---|---|---|---|
| **Digitally enabled growth strategy** | No digitally enabled growth strategy | We react on changing strategies of competitors | There is some understanding of how digital solutions will help us to deliver the firm's objectives | We have started to review our strategy regarding digitally enabled growth. | We have updated our strategy regarding some aspects of digitally enabled growth | We have a consistent digitally enabled innovation & growth strategy in alignment with our resources |
| **Digital Leadership** | No interest of leaders in digitally enabled growth | Reactive leadership. We prefer to firstly see what competitors do. | Leaders recognize potential of digitally enabled growth | Leaders motivate and support people for digital change | Leaders activate employees for digital initiatives | Digital initiatives have high priorityThere is a defined position/role to launch, coordinate and monitor digital initiatives |
| **Digital mindset (attitudes & behaviors)** | Digitally averse | We have some “digital natives” in our firm and they behave accordingly. | People in our firm start thinking about digitalization and managers are developing new digital behaviors. | There is a generally positive attitude towards digitalization and broad development of new behaviors. | Most people in our firm are motivated to seize digital opportunities | Everyone in the firm shares an understanding of our digital vision and has favorable attitudes and behaviors towards digitalization. |
| **Empowered employees** | No empowerment of employees | Some employees have started digital initiatives on their own | We collect ideas of employees for digital innovations | Employees are encouraged to experiment with digital initiatives | Digital initiatives managed by employees have been implemented | Employees are fully empowered to experiment with digital initiatives and implement them. |

## III. Seizing digitally enabled growth potentials

| Dimension | Level 0 | Level 1 | Level 2 | Level 3 | Level 4 | Level 5 |
|---|---|---|---|---|---|---|
| **Digitally enabled business models** | No digitally enabled business models | We have a reactive approach. First, we see what competitors are doing or changing | We are looking for new/improved/innovative business models | We have started to change some components of our business model (e.g. sales channels, value proposition) | Our new business models already contribute to increased revenues | We systematically adapt our business models or create new ones for digital enabled growth, including customer segments, channels, activities/resources and the value proposition |
| **Digital market presence** | No digital market presence | Website not actively managed, some presence in social networks (e.g. Facebook, Twitter, or Linkedin) | Customer-oriented website and presence in several social media. | Actively managed digital market presence through internal resources, collaboration with partners or external service provider. | Our firm has a digital presence and continuously develops different activities to keep ourselves at the top of mind (e.g. digital marketing campaigns, blogs, videos, digital communities, etc.) | Our firm has a broad and integrated *digital* presence across several media through different digital activities. We regularly measure the impact of our digital market presence |
| **Digital Customer experience** | No digital customer experience | Our company has started to interact with customers digitally (e.g.e-mail, online-feedback) | We have at least one well established online channel to deliver customer services and interact with customers. | We actively use customer data for data-driven services. We interact with some customer segments digitally | We mainly interact with customers digitally. Revenues (% of sales) are generated by data-driven services | The firm successfully interacts with (potential) customers and delivers customer service across multiple digital channels, an important part of revenues is generated by data-driven (online) services (% of revenues) |
| **Agile implementation/deployment of digitalization initiatives** | No projects neither methodologies for digital opportunities | Some digital projects have been started, but without using any specific methodology. | Some digital projects have been started and they are following a specific methodology | Digital projects have been started and they have included some techniques to quickly respond to customers' needs and requirements | Some digital projects are based on agile methodologies | The organization has adopted agile methodologies to develop its digital initiatives and this is managed based on a structured method |

## IV. Managing resources for digital transformation

| Dimension | Level 0 | Level 1 | Level 2 | Level 3 | Level 4 | Level 5 |
|---|---|---|---|---|---|---|
| **Digital skills & Learning** | No digital skills. No training for the development of digital skills. | Low level of digital skills and/or low investment to develop digital capabilities. | Adequate level of digital skills in few relevant fields or business areas. | Adequate level of digital skills in some relevant fields or business areas | Significant level of digital skills in most relevant fields or business areas. Specific program for the development of digital skills. | Employees possess all necessary digital skills, which are updated regularly through a formal learning and development program. |
| **Digital processes** | No digitalization of processes | Digitalization of processes is planned | Process digitalization is under way | Few internal processes and interactions with external partners already digitalized | Many internal processes and interactions with external partners already digitalized | Most of processes and interactions with external partners have been digitalized and a formal managerial process to monitor them is in place |
| **Digital technology & security** | Only minimum digital technology in place. Very limited IT security measures | First attempts to adopt digital technology. Regular backup of data, up-to-date antivirus software, further security solutions planned. | Digital technology already adopted in few areas or processes. Few IT and cybersecurity measures are planned | Digital technology starts to be considered a critical enabler for business strategy. Some IT & cybersecurity measures have been implemented. | State-of-the art digital technology supports critical processes and the business strategy; IT security is ensured for the most critical areas. | Effective technology planning, deployment, integration and use to support the digital enabled business. Comprehensive IT security solutions have been implemented for all relevant areas. There is a cybersecurity plan in place. |
| **Digital investments** | No investments related to digitalization | Initial investments in one area or process | Low level of investment on digital initiatives | Medium level of investment on digital initiatives | Significant level of investment on digital initiatives in specific business areas | High level of investment on digital initiatives on all areas of the company based on an integrated business strategy and digital plan |
